# Cultural Determinants of Formal and Informal Volunteerism Among Asian Older Adults and Other Ethnoracial Groups

**DOI:** 10.1093/geroni/igaf122.442

**Published:** 2025-12-31

**Authors:** Patrick Ho Lam Lai, Christina Matz

**Affiliations:** The University of Oklahoma, Moore, Oklahoma, United States; Boston College, Chestnut Hill, Massachusetts, United States

## Abstract

**Background:**

Volunteerism is a key component of productive aging, yet little is known about the cultural influences shaping formal and informal volunteerism among older Asian Americans. This study explores the role of socioeconomic resources and culture-related factors in explaining ethnoracial disparities in formal and informal volunteerism, with a focus on older Asian Americans.

**Methods:**

Using cross-sectional pooled data from the 2004-2020 waves of the Health and Retirement Study (HRS), logistic regression models examined ethnoracial differences in formal and informal volunteerism among adults aged 50-75. The study incorporated cultural indicators (e.g., family composition, emotional support, religious behavior, and immigration status) alongside socioeconomic factors.

**Results:**

Socioeconomic resources (education and income) partially explained volunteerism differences, but cultural factors—particularly religious behavior, family dynamics, and immigration status—further clarified disparities. Differences between Asians and Whites or Blacks diminished after accounting for cultural factors, while gaps with Hispanics persisted.

**Conclusions:**

This study underscores the importance of cultural factors in understanding ethnoracial disparities in volunteerism. Findings suggest that culturally informed interventions are needed to promote productive aging among Asian Americans and other ethnoracial groups. Future research should expand on these findings with more comprehensive cultural indicators and subgroup analyses.

